# Langevin dynamics simulations of charged model phosphatidylinositol lipids in the presence of diffusion barriers: toward an atomic level understanding of corralling of PIP_2_ by protein fences in biological membranes

**DOI:** 10.1186/s13628-014-0013-3

**Published:** 2014-11-26

**Authors:** Kyu Il Lee, Wonpil Im, Richard W Pastor

**Affiliations:** Department of Molecular Biosciences and Center for Bioinformatics, The University of Kansas, Lawrence, KS USA; Laboratory of Computational Biology, National Heart, Lung, and Blood Institute, National Institutes of Health, Bethesda, MD USA

**Keywords:** Phosphotidylinositol 4,5-*bis*phosphate, Protein fence, Diffusion relaxation, Actin, Human septin, Yeast septin

## Abstract

**Background:**

The polyvalent acidic lipid phosphatidylinositol, 4,5-*bis*phosphate (PIP_2_) is important for many cellular functions. It has been suggested that different pools of PIP_2_ exist in the cytoplasmic leaflet of the plasma membrane, and that such pooling could play a role in the regulation of PIP_2_. The mechanism of fencing, however, is not understood.

**Results:**

This study presents the results of Langevin dynamics simulations of PIP_2_ to elucidate some of the molecular level considerations that must be applied to models for fencing. For each simulation, a pool of PIP_2_ (modeled as charged spheres) was placed in containments with boundaries modeled as a single row of rods (steric or electrostatic) or rigid protein filaments. It is shown that even a small gap (20 Å, which is 1.85 times larger than the diameter of a PIP_2_ sphere) leads to poor steric blocking, and that electrostatic blockage is only effective at very high charge density. Filaments of human septin, yeast septin, and actin also failed to provide adequate blockage when placed on the membrane surface. The two septins do provide high blockage consistent with experiment and with phenomenological considerations of permeability when they are buried 9 Å and 12 Å below the membrane surface, respectively. In contrast, burial does not improve blockage by the “arch-shaped” actin filaments. Free energy estimates using implicit membrane-solvent models indicate that burial of the septins to about 10 Å can be achieved without penetration of charged residues into the hydrophobic region of the membrane.

**Conclusions:**

These results imply that a functioning fence assembled from protein filaments must either be buried well below the membrane surface, have more than a single row, or contain additional components that fill small gaps in the filaments.

**Electronic supplementary material:**

The online version of this article (doi:10.1186/s13628-014-0013-3) contains supplementary material, which is available to authorized users.

## Background

Phosphatidylinositol 4,5-*bis*phosphate (PIP_2_) participates in numerous cellular processes such as generation of second messengers, activation of ion channels, endocytosis, and exocytosis [1,2]. Although PIP_2_ occupies only ~2% of the phospholipids on the inner leaflet, there is experimental evidence that the local concentration is significantly enhanced in the regions where exo-, endo-, and phagocytosis occur [1,2]; in the case of the forming phagosome, the local concentration is increased about 3-fold to ~6%. More generally, the pooling of PIP_2_ into specific compartments could allow it to participate in many different cellular functions.

Two different mechanisms of PIP_2_ pooling have been proposed: protein-fencing and protein-binding. In the so-called “protein-fence” hypothesis [3,4], membrane-bound or transmembrane proteins form a boundary that greatly hinders escape of PIP_2_ from the corral. Protein fences have been implicated in restriction in diffusion of membrane proteins and lipids [5]. The alternative “protein-binding” hypothesis, or “reduced diffusion constant hypothesis”, is that peptides or proteins *within* the corral bind PIP_2_, which substantially reduces its diffusion constant, and thereby prevents its escape [6]. For example, PIP_2_ could be bound through simple electrostatic interactions to clusters of basic residues, which exist on many proteins involved in endo-, exo-, and phagocytosis, such as syntaxin and the MARCKS protein [7,8]. Recent measurements of PIP_2_ diffusion in nascent phagosomes by Golebiewska et al. [4,9] have provided unambiguous support for the protein-fence hypothesis in at least this system; diffusion within the corral is similar to other regions in the plasma membrane (and not very different from pure lipid liposomes), but diffusion out of the corral is reduced by a factor of at least 100 (1% of free diffusion). The molecular composition of the fence, however, was not determined.

What makes a good fence? An arrangement of actin filaments would seem to be a suitable candidate. Actin lies on the surface of the nascent phagosomes noted above and is highly negatively charged, as is PIP_2_. Hence, an unbroken row of actin filaments could in principle provide both steric and electrostatic barriers to PIP_2_ diffusion. However, fencing remained after actin was experimentally removed from nascent phagosomes, and computer simulations of PIP_2_-like charged spheres on a membrane surface indicated essentially unimpeded diffusion through an atomic-level model fence composed of actin [4].

This paper extends the aforementioned simulations to evaluate both rod-like and atomic models of protein fences. The PIP_2_ molecules are represented as charged spheres and simulated by Langevin dynamics (LD), which involves generating stochastic trajectories for individual particles consistent with the Langevin equation [10,11]. LD is a close variant of Brownian dynamics [12,13] and the results of the simulations (ratios of relaxation times) would be similar for the two methods [14]. The rod-like fences consist of a row of spheres each comparable to the size of PIP_2_. Steric interactions are isolated by placing the rod-like fences with different sizes of gaps (missing spheres) on the diffusion plane. Electrostatic interactions are probed by charging the spheres (either negatively or positively) and raising the row above the plane to eliminate steric interactions.

While the results of these simulations could be obtained using diffusion equations, a simulation-based solution is pedagogical and provides insight into more complex shapes and arrangements. In addition, the atomic-level particle simulations were performed with actin and two other fence candidates, human septin and yeast septin, at different levels of burial with respect to the diffusion plane. All-atom simulations suitable for studying these systems are not presently possible in terms of time and length scales. Hence, the protein fence is represented by a field on a grid and assumed to be rigid; water and all other membrane components are treated implicitly; i.e., only the PIP_2_-like spheres are simulated. For simplicity, only the single-row fence is considered and the possibility that peptide or other membrane components can bind to the protein filaments and fill in gaps is not explored.

By way of outline, the details of the modeling and simulation are described in the following section. The Results and Discussion section presents and analyzes the decay functions obtained from the simulations, and relates them with the experimental permeability.

## Methods

### Langevin dynamics of PIP_2_

**Figure 1:**
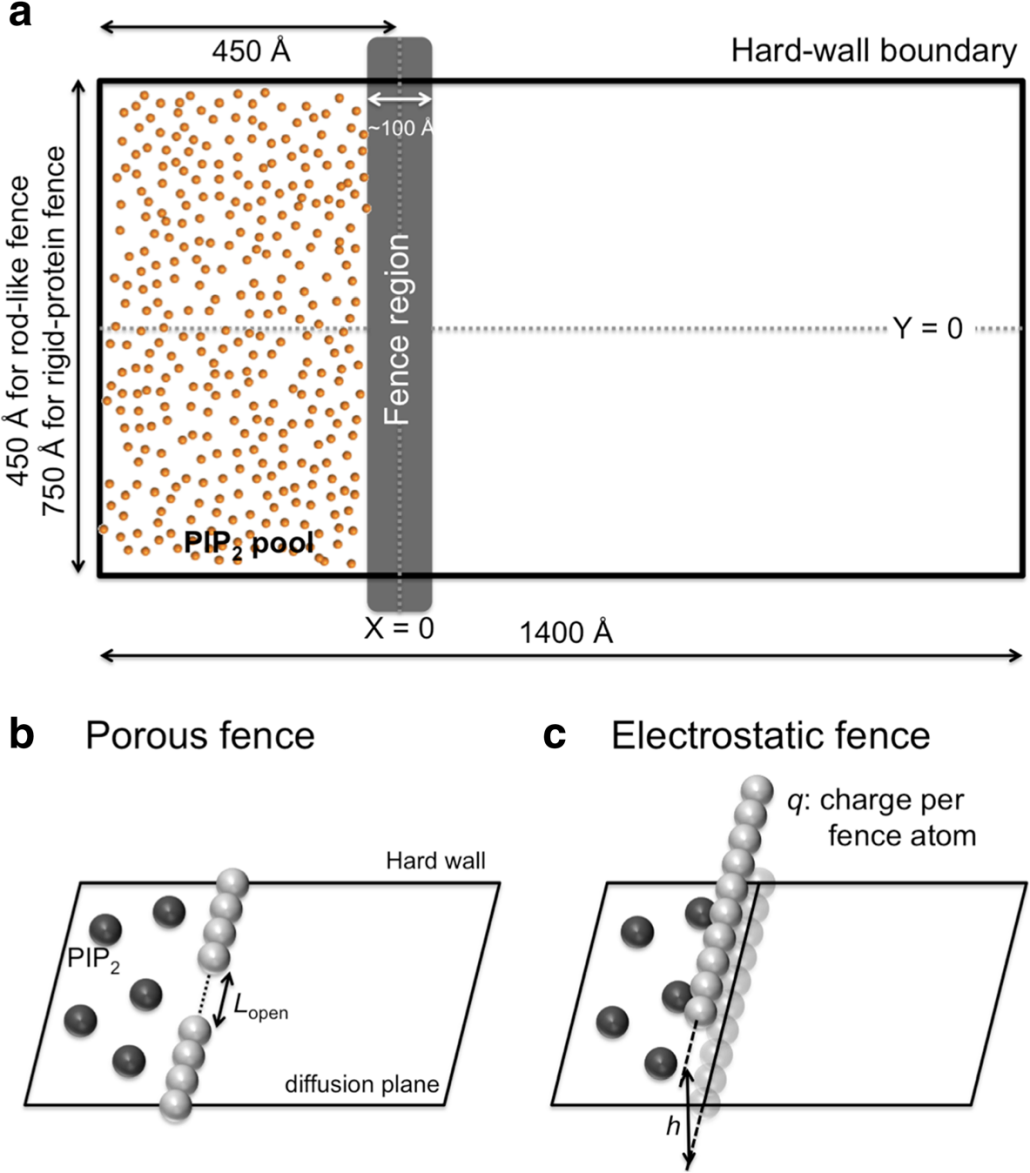
**Schematic representation of the Langevin dynamics system. (a)** Schematic representation of the simulation systems. Each system is a rectangular box with hard-wall boundaries and a fence along the Y-axis centered at X = 0. PIP_2_ molecules are modeled as charged spheres and initially placed inside the pool (X < 0) at a concentration of 6%; the 329 PIP_2_ from one of the septin simulations are shown explicitly (see Figure S3 for snapshots at later times). (B-C) Schematic representations of two types of rod-like fences, with PIP_2_ and fence particles enlarged for clarity **(b)** A porous fence is generated from uncharged blocking atoms on the diffusion plane with a variation in the fence opening length (*L*_open_). **(c)** An electrostatic fence is generated from the single-lined charged atoms placed above the diffusion plane with a variation in the height (*h*) from the diffusion plane and charge per atom (*q*).

The general simulation system is depicted in Figure 1a. PIP_2_ is modeled as a charged sphere (*q* = −4*e*, Lennard-Jones parameters *ε*_LJ_ = 0.5 kcal/mol and *r*_min_/2 = 5.4 Å, and mass *m* = 1,043 amu) and confined to the diffusion plane (Z = 0, XY-plane) by a planar harmonic restraint potential with a force constant of 0.6 kcal/mol/Å^2^. The diffusion plane is located 5 Å above the membrane surface; this is the separation of the phosphates on the inositol ring of PIP_2_ and the phosphate plane of the bilayer obtained from molecular dynamics simulations [15,16].

The systems are enclosed by hard wall boundaries modeled by a harmonic restraint potential with a force constant of 100 kcal/mol/Å^2^ with XY dimensions of 1,400 Å (−475 Å < X < 925 Å) × 1,000 Å for rod-like steric fence, 1,400 Å × 450 Å for rod-like electrostatic fence, and 1,400 Å × 750 Å for the protein fences. Fences were located parallel to the Y-axis at X = 0 (Figure 1a). The “corral” extends from −475 Å < X < 0 Å for all the systems. Simulations were initialized with 438, 197, and 329 PIP_2_-like spheres in the rod-like steric, rod-like electrostatic, and protein corrals (X < 0), respectively; these values correspond to the 6% concentrations of PIP_2_ if restricted inside the corral or 2% if distributed uniformly in the whole PIP_2_-accessible region. LD simulations were carried out with a position independent collision frequency γ = 1 ps^−1^, temperature *T* = 300 K, and a time step of 5 fs. The PIP_2_ spheres were equilibrated for 25 ns and constrained to remain in the corral, and then simulated for 10.0 μs without constraints. Different random seeds were used to generate 20 independent trajectories for the rod-like fences and 30 for the protein fences. The diffusion constant *D* = *k*_*B*_*T*/*mγ* = 2.4 ×10^−5^ cm^2^/s is over 10 times faster than the experimental value [4]. However, because only the *ratios* of hindered to free diffusion are considered, this large value of *D* does not alter the conclusions, and is computationally efficient. Note also that *D* is the same throughout the region, although the *effective* diffusion constant may be enhanced or retarded in the vicinity of the fence, and is larger in the beginning of the trajectory arising from repulsion of the changed particles in the corral.

Lastly, Brownian dynamics would have been equally acceptable for this study, but it is not available in CHARMM [17], the simulation package used.

### Rod-like fences

As noted in the Background section, the rod-like models were simulated to quantify the general blockade characteristics for purely steric and electrostatic potentials. Steric fences (Figure 1b) of length *L* = 1,000 Å were developed by placing impenetrable spheres with no charge and gaps specified by the fence opening length (*L*_open_) on the diffusion plane. Simulations were performed with *L*_open_ = 10, 20, 30, 40, 50, 100, 200, and 500 Å to model increasingly porous fences, and *L*_open_ = 1,000 Å to model free diffusion. The electrostatic fences (Figure 1c) are single-lined charged spheres with charges (*q* = ±0.05*e*, ±0.10*e*, ±0.50*e*, and ±1.00*e*) and heights above the diffusion plane (*h* = 2, 5, 7, and 10 Å).

### Protein fences

**Figure 2:**
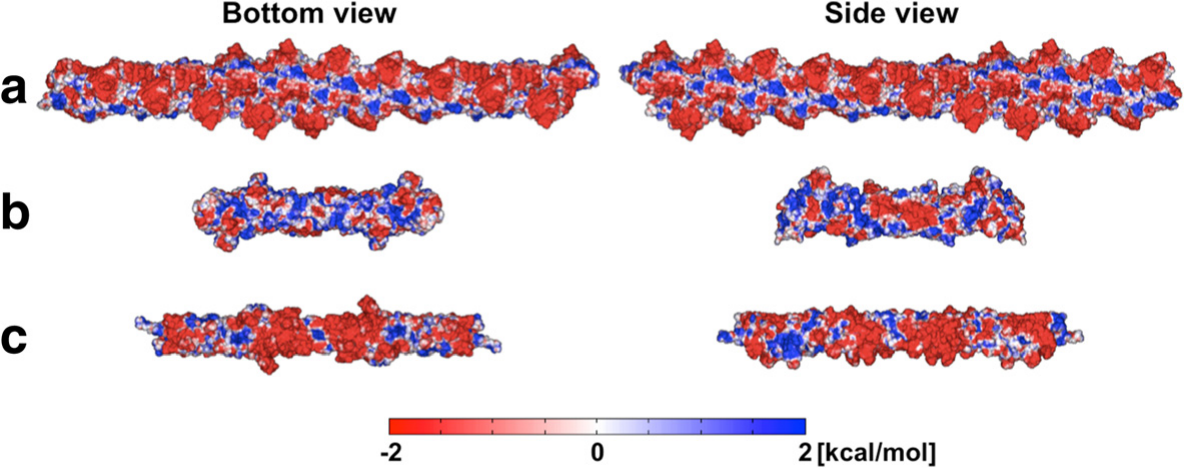
**Electrostatic potential on the molecular surfaces. (a)** Actin, **(b)** Human septin, and **(c)** Yeast septin. Half of the biological units of the septins (Sept7-Sept6-Sept2 for human septin and Cdc11-Cdc12-Cdc3-Cdc10 for yeast septin) are shown here for simplicity. The blue regions shown in the bottom view of the septins are the polybasic regions, which are assumed to be the binding surface to the membrane in the simulation.

PIP_2_ pools in cells are several hundred nanometers on a side [4,9] and appear to be bounded by long filamentous structures. We thereby assume that corral-building proteins have two physical properties. First, the fence proteins bind to or interact strongly with the anionic lipid membrane and/or PIP_2_, and second, the fence is built from filament formation of its subunits. Among peripheral membrane proteins satisfying these two conditions, actin and septins (human and yeast) were selected as protein fence candidates (Figure 2). Actin is one of the major components of the mammalian cytoskeleton. Septin has been reported to colocalize with actin filaments. All three proteins have been found near PIP_2_-abundant regions [18-20].

An actin fence was built from an X-ray fiber based model (PDB:2ZWH) of Oda et al. [21] and by adding additional monomers with 166.4° rotations and 27.6 Å translations along the fence axis to generate an actin filament. A human septin fence was built from the hexameric biological unit (Sept7-Sept6-Sept2-Sept2-Sept6-Sept7) in PDB:2QAG [22]. Missing coordinates of some residues in PDB:2QAG were reconstructed using I-TASSER [23], a protein structure prediction tool. The model structure was then aligned to the original PDB to determine the best-fit structure. Additional biological units were placed by 253.2 Å translations along the fence axis to generate a human septin filament.

There is no reported PDB structure for octameric yeast septin, but the full sequence and the octameric biological unit (Cdc11-Cdc12-Cdc3-Cdc10-Cdc10-Cdc3-Cdc12-Cdc11) of the protein is available. Atomic coordinates of the full sequence were generated using I-TASSER and the best-fit structure was selected from the alignment to the human septin model. The sequence identity between the subunits of yeast septin and human septin ranges from 32% to 38% (comparison in Protein BLAST [24]), which supports the similar structures to each other. Additional biological units were placed by 338.7 Å translations along the fence axis to generate a yeast septin filament.

The resulting septin structures form a long filamentous rod. The binding surfaces of the septins to the lipid membrane are assumed to be the one with the polybasic region that is conserved among septin family and its subunits (Additional file 1: Figure S1), thereby electrostatic attraction is expected to enhance the binding of the septins to the anionic lipid [18,19]. The septins contain long C-terminal extensions that are believed to play a key role in binding with the other septins to make parallel filament bundle [25]. The C-terminal extensions were excluded here because they appear to contribute little to the single filament formation and binding of the septins to the membrane.

### Continuum electrostatics based effective potentials

The interactions between PIP_2_-like charged spheres and the environments (water, membrane, and protein) were approximated by static electrostatic (*ϕ*^PHIX^) and repulsive core (*U*^CORE^) potentials [26]. Using the Poisson-Boltzmann PBEQ module [27,28] in CHARMM [17], *ϕ*^PHIX^ was calculated with an implicit membrane with a thickness of 36 Å and a dielectric constant of 2, the solution region with a dielectric constant of 80 and a salt concentration of 150 mM, and the proteins or artificial fence atoms with the optimized PB atomic radii [29] and a dielectric constant of 2. The electrostatic potential was first calculated with a coarse grid (grid-spacing of 1.0 Å), the result of which was then used to set the potential on a finer grid (grid-spacing of 0.5 Å). The PB radii augmented by 4.16 Å were used to setup the molecular surface by which a core repulsion potential map was built. *U*^CORE^ was set to zero in all PIP_2-accessible_ regions (outside proteins or artificial fence atoms) and 100 kcal/mol otherwise. The electrostatic and repulsive core potentials were stored on a grid (2,801 × 2,001 × 61 for the rod-like steric fence, 2,801 × 901 × 61 for the rod-like electrostatic fence, and 2,801 × 1,501 × 61 for the protein fence with a grid-spacing of 0.5 Å). Therefore, in this system representation, PIP_2_ molecules are the only explicitly simulated entities and the PIP_2_ energy is calculated by

1 $$ {U}_{\mathrm{PIP}2}\left({\mathrm{r}}_i\right)={\displaystyle \sum_{j\ne i}\left[{\varepsilon}_{ij}\left\{{\left({r}_{\min }/{r}_{ij}\right)}^{12}-2{\left({r}_{\min }/{r}_{ij}\right)}^6\right\}+\frac{q_i{q}_j}{80{r}_{ij}}\right]}+{U}^{CORE}\left({\mathrm{r}}_i\right)+{q}_i{\varPhi}^{\mathrm{PHIX}}\left({\mathrm{r}}_i\right) $$

Note that *ϕ*^PHIX^ and *U*^CORE^ were computed once before the LD simulations, and their energies and forces were calculated using the 3rd-order B-spline interpolation during the simulations [30,31]. The preparation of the simulation system (PDB manipulation), calculation of the potential maps (PBEQ module), and LD simulations were performed using CHARMM [17] and CHARMM-GUI (http://www.charmm-gui.org) [32].

### Analysis

The depletion of PIP_2_ population, i.e., the concentration decrease in the PIP_2_ pool from the initial 6%, characterizes the PIP_2_ diffusion through the fence. The number of particles in the pool was evaluated as a function of time and averaged over the independent runs. All decay curves were well fit by the stretched exponential function:

2 $$ C(t)=\left({C}_0-{C}_{\infty}\right) \exp \left[-{\left(t/\tau \right)}^{\beta}\right]+{C}_{\infty } $$

where *C*_∞_, *τ*, and *β* are iteratively determined from the simulation results with *C*_0_ = 6%. The average relaxation time from the stretched exponential curve is given by

3 $$ <\tau >=\frac{\tau }{\beta}\cdot \varGamma \left(1/\beta \right) $$

where Γ is the gamma function defined as $\varGamma (x)={\int}_0^{\infty }{t}^{x-1}{e}^{-t}dt$. The retardation of diffusion is then quantified by the concentration relaxation time ratio *ξ* = < *τ*_free_ >/< *τ* >, where < *τ*_free_ > is the relaxation time in the absence of a barrier, i.e., free diffusion.

The potential of mean force (PMF) was calculated from position dependent concentrations of PIP_2_ after equilibrium had been reached [33]:

4 $$ {W}_{1\mathrm{D}}(x)={k}_{\mathrm{B}}T\cdot \ln \left[{C}_{\mathrm{eq}}(x)/{C}_{\mathrm{ref}}\right] $$

where *C*_ref_ is set to the bulk concentration under equilibrium (2%). The PMF represents the potential profile in the presence of PIP_2_ molecules in the equilibrated simulation system.

## Results and Discussion

### Rod-like fences

Additional file 1: Figure S2 shows the characteristics of the rod-like fences. The steric fences range from nearly continuous to 50% breached (Additional file 1: Figure S2A), and the electrostatic fences from strongly negative to strongly positive (Additional file 1: Figure S2B). The positive (attractive potential well) and negative charges (repulsive potential barrier) yield symmetric electrostatic potential profiles (Additional file 1: Figure S2C).

**Figure 3:**
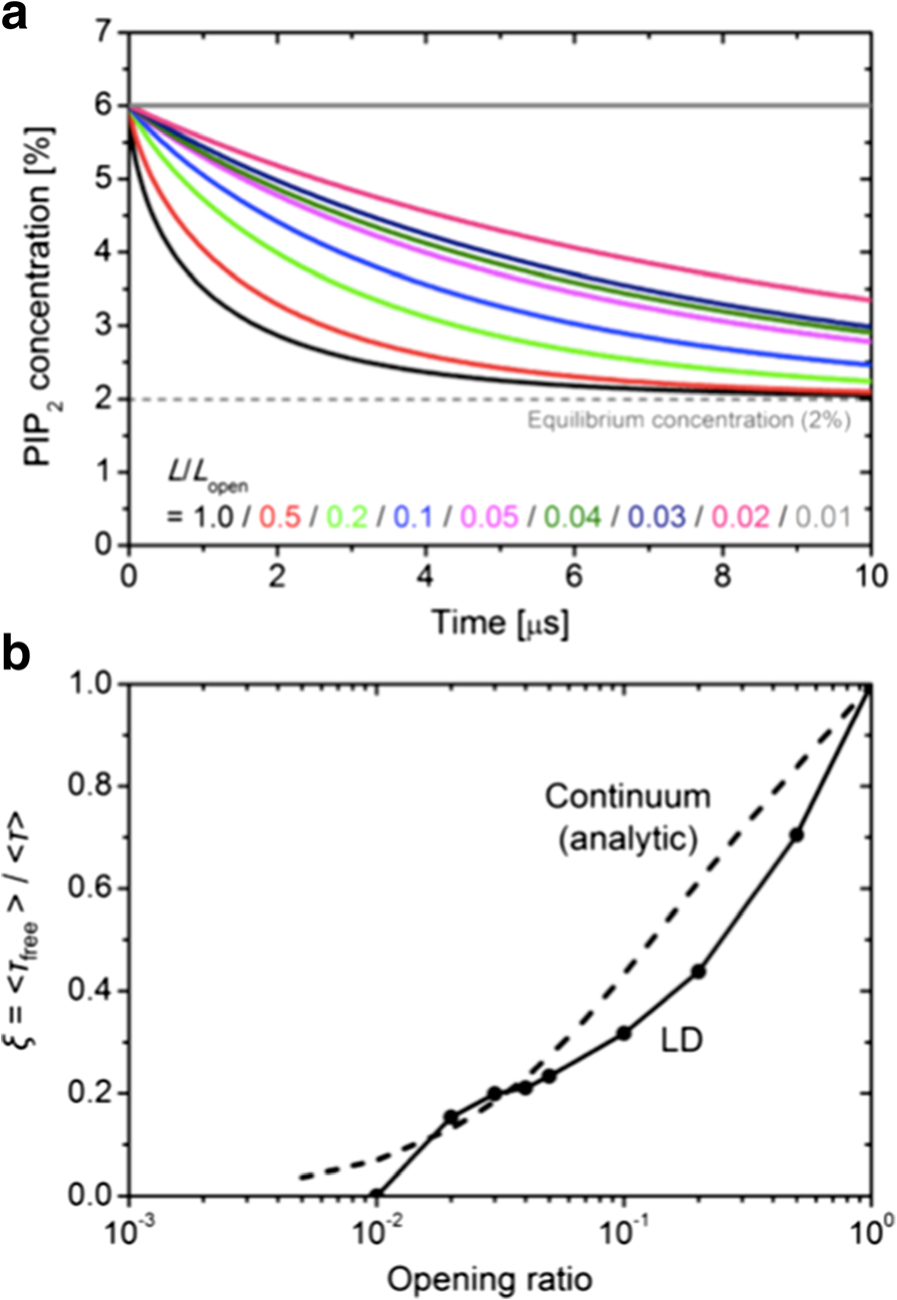
**Results for rod** **-** **like steric fences. (a)** Time-series of PIP_2_ concentration in the pool with different fence opening ratio (*L*_open_/*L*) with *L* = 1,000 Å. **(b)** Ratio of the relaxation time with respect to free diffusion (<*τ*_free_ > for *L*_open_ = *L*). The dotted line labeled as Continuum is obtained from the one-dimensional diffusion of continuum matter (unpublished).

The retardation of PIP_2_ diffusion through the fence was estimated by the relative change of the relaxation time with respect to the free diffusion case (*L*_open_ = 1,000 Å): *ξ* = < *τ*_free_ >/< *τ* >. The time-series of the PIP_2_ concentration inside the pool (depletion) for the porous fences and its fitted curves are shown in Figure 3a and the resulting relaxation times with respect to free diffusion (*ξ* defined above) are shown in Figure 3b (see Additional file 1: Figure S3 for representative snapshots from the trajectory). There is little retardation of diffusion for the larger gaps, and even for *L*_open_ = 20 Å (which is 1.85 times larger than the size of PIP_2_) the relaxation times with respect to free diffusion are only reduced by a factor of 6. Hence, the gap in the fence should be very close to the size of PIP_2_ to reproduce the experimentally observed factor of 100 reduction in diffusion [4]. This result highlights ineffectiveness of purely steric repulsion for blocking diffusion; once a diffusing particle is in the vicinity of a gap, it has multiple opportunities to escape through the gap due to its stochastic motions. (For ballistic trajectories, passage through a barrier is simply proportional to the fraction of the barrier that is not blocked.) Additionally, as deduced from a comparison with a continuum calculation for free diffusion, repulsion between the PIP_2_ particles (*q* = −4*e*) in the corral provides as the second driving force for dispersal.

**Figure 4:**
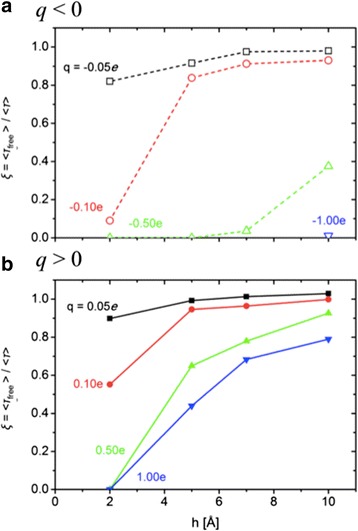
**Concentration relaxation time ratios for rod** **-** **like electrostatic fences.** Concentration relaxation time ratio for the electrostatic fence simulations, calculated from Figure 4: **(a)** *q* < 0 and **(b)** *q* > 0. < *τ*_free_ > refers to the free diffusion case (*q* = 0).

Turning to the electrostatic models, the concentration relaxation time ratios *ξ* for assorted cases are plotted in Figure 4 (the depletion curves in Additional file 1: Figure S4). The general trends are not surprising. For both negatively and positively charged rods, blockage increases with increasing charge and decreasing height above the surface. However, the negative rods are more effective at the same charge magnitude and height. The difference arises because negatively charged rod directly repels PIP_2_ (there is an energy barrier at X = 0, directly under the rod), while the positively charged rod attracts PIP_2_ (there is an energy well at this position). In particular, from Figure 4, complete blockade of PIP_2_ diffusion is observed for *q* = −1.0*e* when *h* ≤ 7 Å, *q* ≤ −0.5*e* when *h* = 5 Å, and |*q*| ≥ 0.5*e* when *h* ≤ 2 Å. These conditions correspond to electrostatic energy barriers larger than 7.5 kcal/mol and energy wells deeper than 12.5 kcal/mol, as calculated for the interaction of an individual PIP_2_ with the rod.

**Figure 5:**
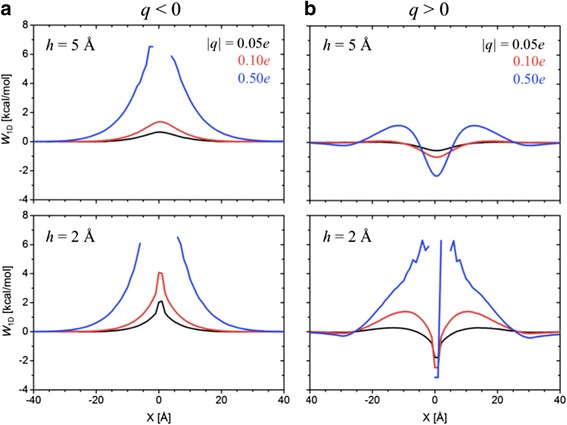
**Potentials of mean force for rod** **-** **like electrostatic fences.** Potentials of mean force (PMFs) for *h* = 5 Å and 2 Å, calculated from equilibrium simulations with the electrostatic fences: **(a)** *q* < 0 and **(b)** *q* > 0.

The explanation for the difference requires consideration of the interactions between PIP_2_. The potential of mean force (PMF) provides the entry point for this analysis. Figure 5 plots the PMFs calculated from equilibrium simulations of initial uniform 2% PIP_2_ distribution using Eq. (4). Negative charges on the rod lead to a nearly impermeable fence when the PMF barrier is larger than 6.5 kcal/mol. The difference between the electrostatic potential barrier of 7.5 kcal/mol (at X = 0) and the PMF barrier of 6.5 kcal/mol is due to the lack of PIP_2_ sampling near X = 0. Positive charges result in accumulation of PIP_2_ in the potential well, which, in turn, can exert a potential barrier for the other PIP_2_ to enter and then move through the fence; i.e., there is both a well under the rod and a barrier on each side. A nearly complete blockade occurs when the trapped PIP_2_ in the potential well below the fence exerts an energy barrier of around 6.5 kcal/mol to the PIP_2_ outside of the fence (Figure 5, *h* = 2 Å).

### Protein fences

**Figure 6:**
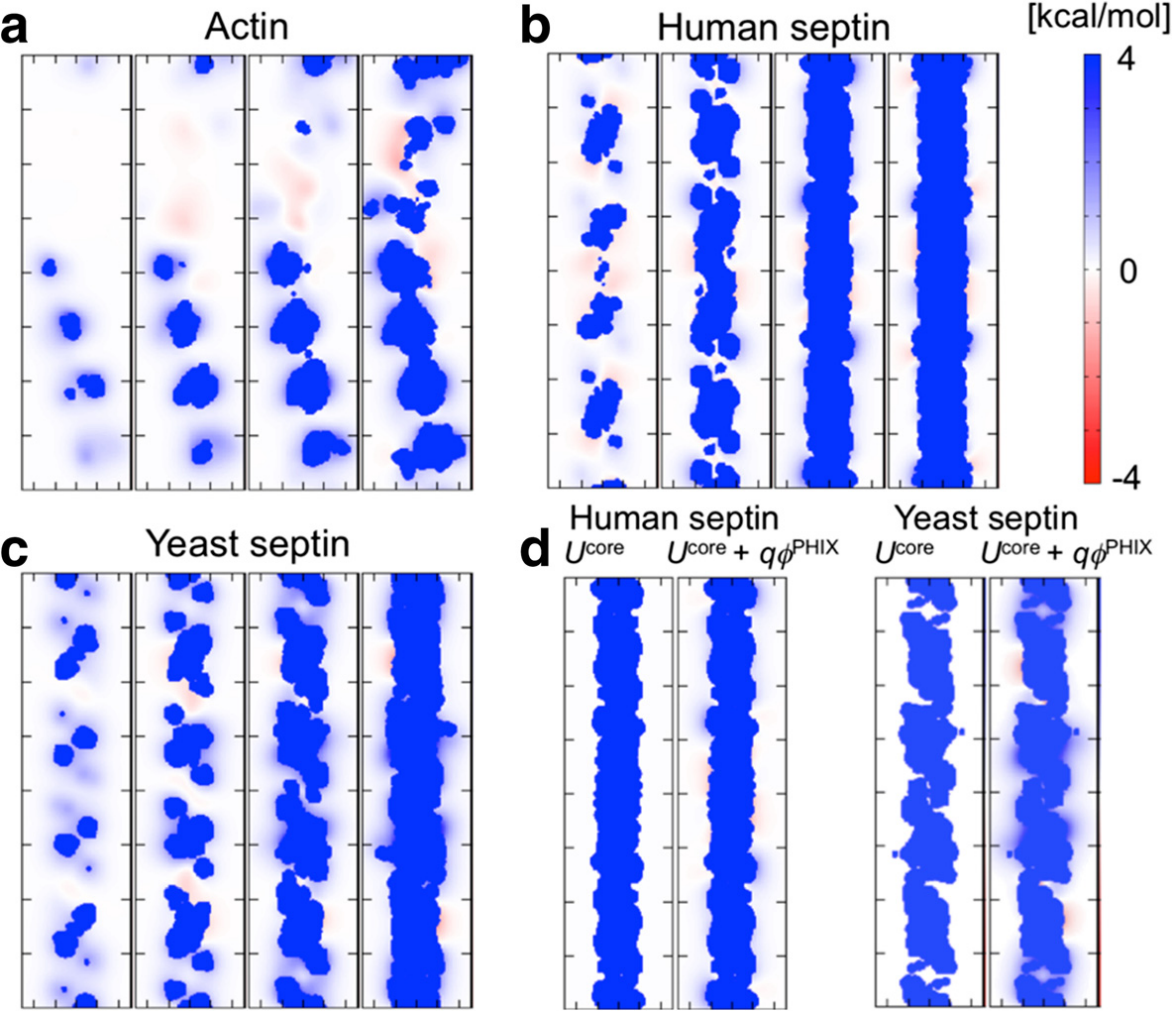
**Potential maps of the rigid** **-** **protein fences.** The potential maps of the rigid-protein fences at four depths (Z_min_) of protein binding to the lipid membrane, Z_min_ = 0, −5, −10, and −15 Å (from left to right): **(a)** Actin, **(b)** Human septin, and **(c)** Yeast septin. **(d)** Comparison of the steric potential (*U* ^CORE^) and the steric plus electrostatic potential (*U* ^CORE^ + *q*ϕ^PHIX^) for human septin with Z_min_ = −9 Å and yeast septin with Z_min_ = −12 Å.

**Figure 7:**
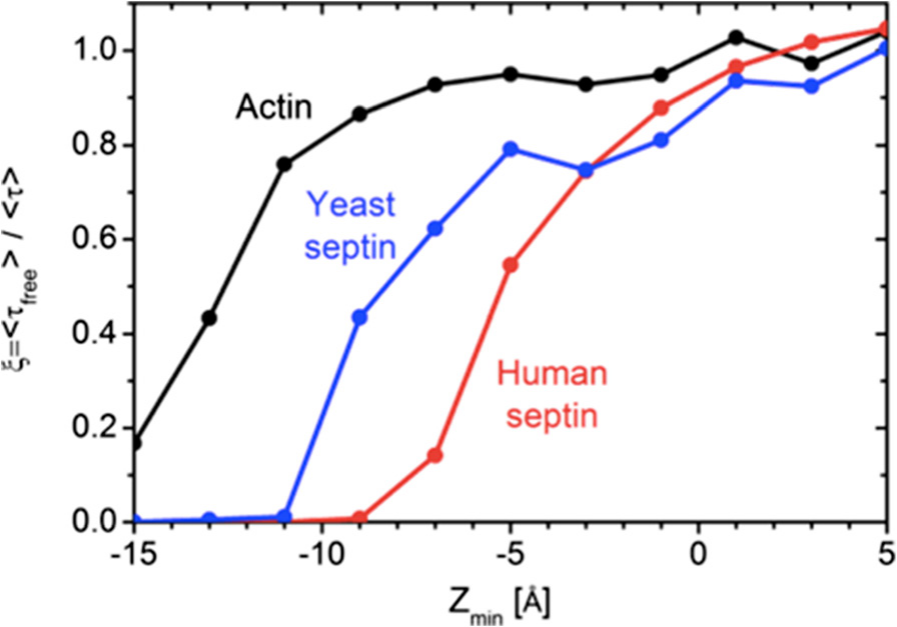
**PIP**_**2**_ **depletion for the rigid** **-** **protein fences.** Comparison of the concentration relaxation time ratio for the rigid-protein fences at different burial depths (Z_min_).

Figure 2 shows the electrostatic potentials from the protein charges projected on the molecular surfaces of (A) actin, (B) human septin, and (C) yeast septin. The polybasic amino acids of the septins (Additional file 1: Figure S1) are assumed to be the binding surfaces to the membrane (blue regions in “bottom view”). These electrostatic potentials are combined with the surrounding dielectrics and steric repulsive potentials from the proteins to develop the total potentials. Figure 6 maps the potentials for PIP_2_ on the diffusion plane at Z_min_ = 0, −5, −10, and −15 Å, where Z_min_ is the minimum location of the protein atoms to the membrane surface (Z = 0; the lipid phosphate plane). Figure 6d shows the different components of the potential at Z_min_ = −9 Å for human septin and Z_min_ = −12 Å for yeast septin where a complete blockade is observed from trajectory analysis (Figure 7). Importantly, when Z_min_ = 0 Å (at least one protein atom is in contact with the diffusion plane), the steric and electrostatic potentials yield a relatively porous projection on the surface.

The concentration relaxation time ratio *ξ* is plotted as a function of Z_min_ in Figure 7. Consistent from the potential maps in Figure 6, fencing is ineffective when Z_min_ = 0 Å. This result is anticipated from the steric rod models, where even a small gap results in nearly free diffusion (Figure 3). Actin has a pronounced arch-shape (Figure 2a), which leaves unblocked fence even with the deepest burial (Figure 6a, Z_min_ = −15 Å). Consequently, all the actin fence results show significant PIP_2_ escape out of corral (Figure 7) for all burial depths. Septins provide essentially complete blockage (*ξ* ≈ zero) when Z_min_ = −9 Å for the human septin fence and Z_min_ = −12 Å for the yeast septin fence. Specifically, the potential maps calculated at Z_min_ = −9 Å for the human septin fence and Z_min_ = −12 Å for the yeast septin fence (Figure 6d) show the “blanket coverage” required for blockage found in the rod-like fence studies. Interestingly, human and yeast septin block PIP_2_ diffusion in different ways as revealed by the comparison of the steric (*U*^CORE^) and electrostatic (*ϕ*^PHIX^) potentials. *U*^CORE^ from the human septin fence at Z_min_ = −9 Å provides complete coverage. In contrast, narrow gaps in *U*^CORE^ from yeast septin fence at Z_min_ = −12 Å are filled in by *ϕ*^PHIX^ for complete blockade.

**Figure 8:**
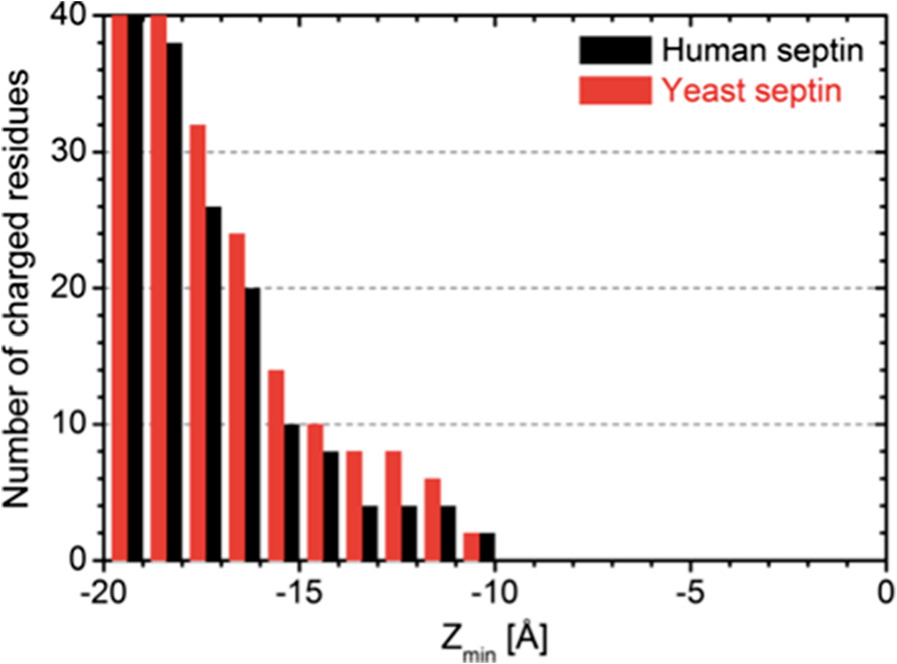
**Number of charged residues inside the hydrophobic region.** Number of charged residues inside the hydrophobic region as a function of the burial depths (Z_min_) of the human and yeast septin fences. The number of charged residues is counted in 1 Å bin along the Z-axis.

Given the preceding simulation results that the septin fences can block PIP_2_ diffusion when buried at certain depths from the membrane surface, the next question is the likelihood of having such burial depths. While it is challenging to quantify the penetration depth of peripheral membrane proteins in both experimental and computational studies, there are three qualitative arguments that such burial depths are possible. First, there is the polybasic region exposed on the putative membrane-binding protein surface, which enhances electrostatic binding to the anionic lipid membrane (Additional file 1: Figure S1). Second, there is abundance in hydrophobic residues (PHE, TYR, and TRP) on the membrane-binding protein surface, especially those with hydrophobic rings. As shown in Additional file 1: Figure S1, both human and yeast septins have hydrophobic residues in the loops near the polybasic region and the other basic residues. These hydrophobic rings can act as binding anchors into the hydrophobic core of the lipid membrane. It has been reported that anchoring of MARCKS effector domain to the lipid membrane is enhanced by the penetration of five PHE rings into the lipid hydrophobic core [8]. Third, there is no penetration of charged residues in the present rigid models into the hydrophobic region even at Z_min_ = −10 Å, i.e., 10 Å below the lipid phosphate plane (Figure 8). Thus, the charged residues can stay in the head region without invoking large free energy penalty due to their burial into the hydrophobic core. However, the remaining 2 Å penetration for yeast septin is not guaranteed in the current rigid-protein fence model. Further (computationally very demanding) studies would be required to explore the influence of the protein conformation change on membrane binding and to determine whether such charged residues can re-orient themselves to avoid the burial penalty.

### Phenomenological treatment of permeability

Let us assume, for simplicity, a square corral of 1 μm per side. From Hilgemann’s analysis [34], we can also assume that there are 100 kinases inside the corral and each kinase produces 100 PIP_2_ molecules per second, hence production rate is of 10^4^ molecules/(μm^2^⋅s). The permeability *P* through a fence region can be defined as *P* = *k* ⋅ *D*/*d*, where *k* is the partition coefficient of PIP_2_ from the bulk membrane into the fence region, *D* is the diffusion coefficient, and *d* is the thickness of the fence region. Assuming *d* = 5 nm and *D* =1 μm^2^/s (in the bulk region [4]), *P* = 200 *k* μm/s. The flux per unit length *F* of PIP_2_ out of the fence can be simplified using Fick’s law as *F* = *P* (*C*_*i*_ − *C*_*o*_), where *C*_*i*_ (from our rigid-protein model, 10^5^ μm^−2^) is the areal concentration of PIP_2_ inside the fence and *C*_*o*_ (3 × 10^4^ μm^−2^) that in the bulk. The steady-state PIP_2_ flux out of the corral can be estimated as *F* = 1.4 × 10^7^*k* / (μm⋅s). As the condition for the corral to act as a barrier maintaining the concentrated pool of PIP_2_, the outward flux should be kept below of the production rate. If the outward flux is only through one side of the square, the partition coefficient *k* should be smaller than 7 × 10^−3^ (= 10^4^ / (1.4 × 10^7^)). In other words, if the fence is leaky to PIP_2_ above this value, the fence would not be an effective barrier to maintain the PIP_2_ pool. This impermeability calculation for an effective protein fence is in good agreement with our simulation results, which predicts virtually complete blockade from *U*^CORE^ or *U*^CORE^ + *q*_PIP2_*ϕ*^PHIX^ barrier (Figures 6d and 7).

## Conclusions

Langevin dynamics simulations of PIP_2_ on a membrane surface with rod-like steric and electrostatic as well as all-atom protein fence models were carried out to determine the general conditions for fencing PIP_2_ and the specific fencing ability of actin, human septin, and yeast septin. Simulations on the model systems indicate that even a small gap leads to ineffective blocking. Likewise, electrostatic blockage is only effective at very high charge density, and is unlikely to play a major role in blockage of PIP_2_ diffusion.

These observations place significant limitations on the abilities of individual proteins to form effective fences. In fact, single filaments of actin, human septin, and yeast septin provided little blockage when placed on the membrane surface. Even burial to 15 Å did not yield significant blocking by actin, as could be anticipated by its pronounced arch-like shape. However, the two septins did provide blockage consistent with experiment when the human septin is buried 9 Å and the yeast septin 12 Å below the membrane surface. Implicit membrane-solvent models indicate that burial to 10 Å can be achieved, though further penetration requires protein conformational changes. All-atom simulations with fully flexible proteins and explicit membrane and solvent will undoubtedly yield further insight to the mechanism of blockage.

Though not simulated here, a fence could be made more effective by adding more rows or more components. For example, if each of three rows provides 80% blockage and the rows are independent, the array leads to more than 99% blockage. Similarly, a small gap in a single row could be filled by a membrane-associated peptide. In closing, it was not the purpose of this study to determine the molecular level mechanism of corralling of PIP_2_ on the cell surface by protein fences. Rather, it was to elucidate some of the molecular level considerations that must be applied to models for fencing, and to stimulate more detailed simulation studies.

## Additional file

Additional file 1: Figure S1. Basic and hydrophobic residues of the septins. Basic (blue: ARG and LYS) residues found in the polybasic regions and hydrophobic residues. with aromatic ring (magenta: PHE, green: TRP, and gray: TYR): (A) Human septin and (B). Yeast septin. **Figure S2.** Potential energy for rod-like fences. (A) Steric repulsive potential energy from the porous fences according to the fence opening. (*L*_open_) with the fence length *L* = 1,000 Å. (B-C) Potential energy from the electrostatic fences according to the charge per fence atom (*q*) and height (*h*) of the charged rod from the diffusion plane with *L* = 450 Å: (B) 2D potential map and (C) 1D profile along the X-axis (diffusion direction, Y = 0). **Figure S3.** Representative snapshots of the PIP2 depletion from the trajectory. Trajectory snapshots for human septin fence with Z_min_ = 8 Å taken at (A) initial configuration (*t* = 0), (B) *t* = 0.1 μs, (C) *t* = 1 μs, and (D) *t* = 5 μs. **Figure S4.** Results for rod-like electrostatic fences. Time-series of PIP2 concentration in the pool with different heights (*h*) of the charged bar from the diffusion plane and atomic charge (*q*): (A) *h* = 10 Å, (B) *h* = 7 Å, (C) *h* = 5 Å, and (D) *h* = 2 Å. The positive fence charge cases are shown in solid lines and negative fence charge cases in dotted lines. Black, red, green, and blue lines are for |*q*| = 0.05*e*, 0.10*e*, 0.50*e*, and 1.00*e*, respectively.
