# Supplementary material for: Langevin dynamics simulations of charged model phosphatidylinositol lipids in the presence of diffusion barriers: toward an atomic level understanding of corralling of PIP2 by protein fences in biological membranes
Source: BMC Biophys. 2014 Nov 26;7:13. doi: 10.1186/s13628-014-0013-3 (PMC4358915; doi:10.1186/s13628-014-0013-3)
Supplement: Additional file 1: Figure S1. — Basic and hydrophobic residues of the septins. Basic (blue: ARG and LYS) residues found in the polybasic regions and hydrophobic residues. with aromatic ring (magenta: PHE, green: TRP, and gray: TYR): (A) Human septin and (B). Yeast septin. Figure S2. Potential energy for rod-like fences. (A) Steric repulsive potential energy from the porous fences according to the fence opening. (L open) with the fence length L = 1,000 Å. (B-C) Potential energy from the electrostatic fences according to the charge per fence atom (q) and height (h) of the charged rod from the diffusion plane with L = 450 Å: (B) 2D potential map and (C) 1D profile along the X-axis (diffusion direction, Y = 0). Figure S3. Representative snapshots of the PIP2 depletion from the trajectory. Trajectory snapshots for human septin fence with Zmin = 8 Å taken at (A) initial configuration (t = 0), (B) t = 0.1 μs, (C) t = 1 μs, and (D) t = 5 μs. Figure S4. Results for rod-like electrostatic fences. Time-series of PIP2 concentration in the pool with different heights (h) of the charged bar from the diffusion plane and atomic charge (q): (A) h = 10 Å, (B) h = 7 Å, (C) h = 5 Å, and (D) h = 2 Å. The positive fence charge cases are shown in solid lines and negative fence charge cases in dotted lines. Black, red, green, and blue lines are for |q| = 0.05e, 0.10e, 0.50e, and 1.00e, respectively. [file 13628_2014_13_MOESM1_ESM.pdf]

Additional file 1

Langevin Dynamics Simulations of Charged Model Phosphatidylinositol Lipids in the Presence of Diffusion Barriers: Toward an Atomic Level Understanding of Corralling of PIP$_2$ by Protein Fences in Biological Membranes

Kyu Il Lee[1], Wonpil Im[1*], Richard W. Pastor[2*]

[1]*Department of Molecular Biosciences and Center for Bioinformatics, The University of Kansas, Lawrence, KS*

[2]*Laboratory of Computational Biology, National Heart, Lung, and Blood Institute, National Institutes of Health, Bethesda, MD*

[*]Corresponding authors

Email addresses:

KIL: macjr@ku.edu

WI: wonpil@ku.edu

RWP: pastorr@nhlbi.nih.gov

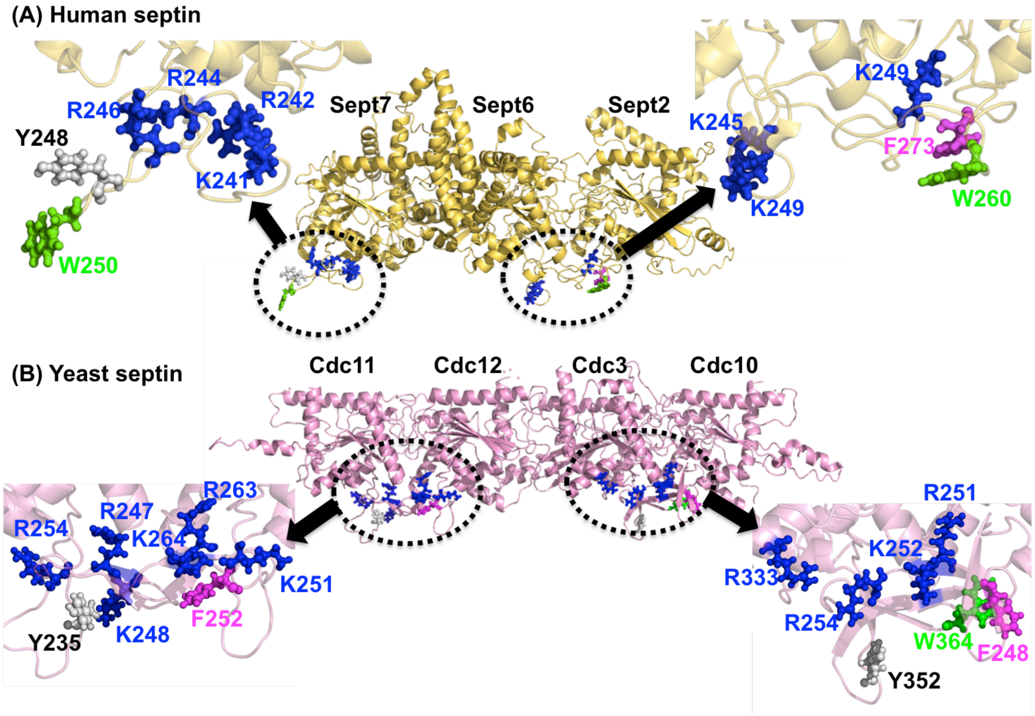

Figure S1 – Basic and hydrophobic residues of the septins

Basic (blue: ARG and LYS) residues found in the polybasic regions and hydrophobic residues with aromatic ring (magenta: PHE, green: TRP, and gray: TYR): (A) Human septin and (B) Yeast septin.

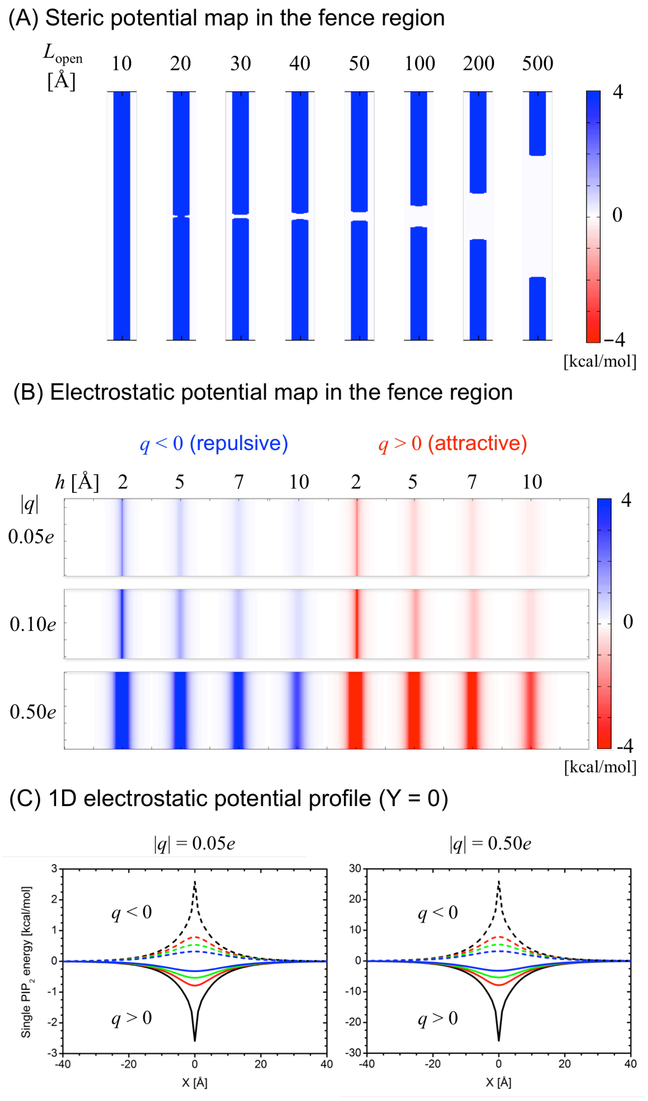

Figure S2 – Potential energy for rod-like fences

(A) Steric repulsive potential energy from the porous fences according to the fence opening (L_{open}) with the fence length L = 1000 Å. (B-C) Potential energy from the electrostatic fences according to the charge per fence atom (q) and height (h) of the charged rod from the diffusion plane with L = 450 Å: (B) 2D potential map and (C) 1D profile along the X-axis (diffusion direction, Y = 0).

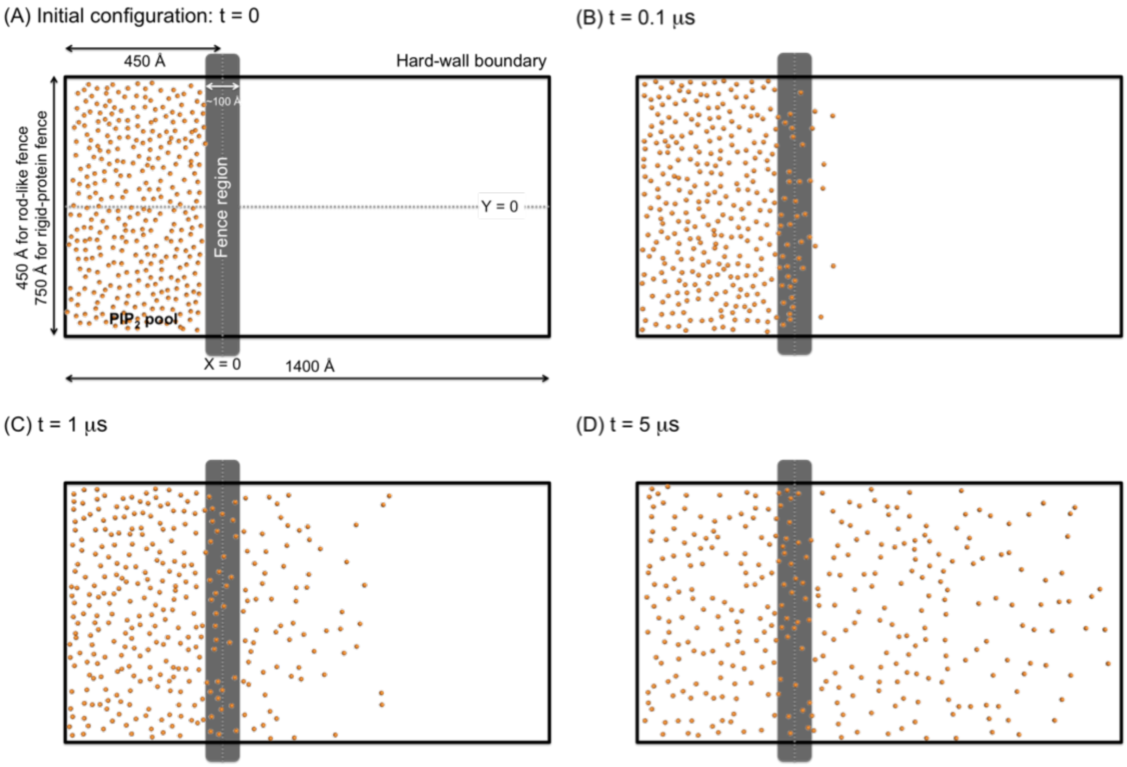

Figure S3 – Representative snapshots of the PIP_2 depletion from the trajectory

Trajectory snapshots for human septin fence with Z_{min} = 8 Å taken at (A) initial configuration (t = 0), (B) t = 0.1 μs, (C) t = 1 μs, and (D) t = 5 μs.

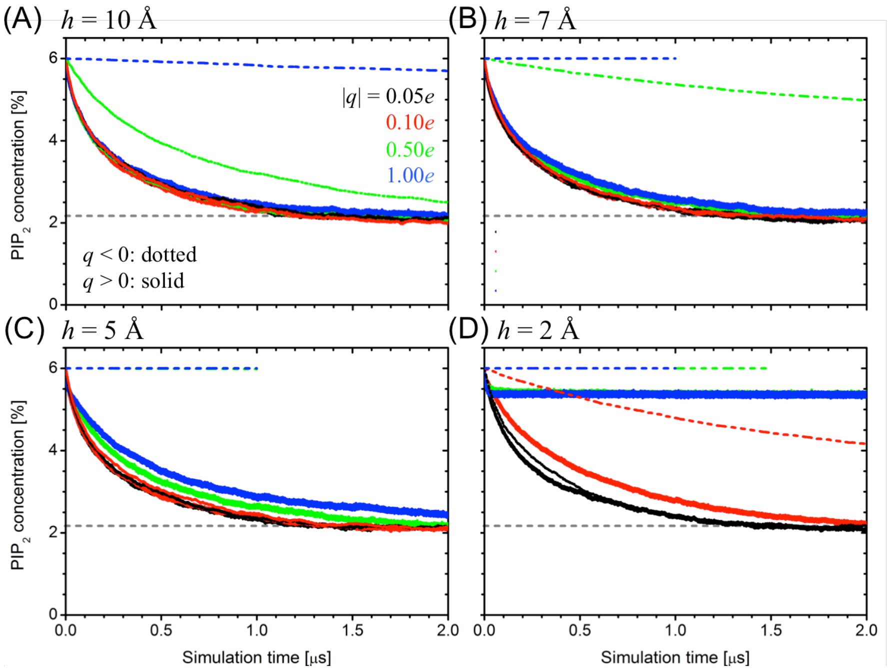

Figure S4 – Results for rod-like electrostatic fences

Time-series of PIP_2 concentration in the pool with different heights (h) of the charged bar from the diffusion plane and atomic charge (q): (A) h = 10 Å, (B) h = 7 Å, (C) h = 5 Å, and (D) h = 2 Å. The positive fence charge cases are shown in solid lines and negative fence charge cases in dotted lines. Black, red, green, and blue lines are for $|q|$ = 0.05e, 0.10e, 0.50e, and 1.00e, respectively.
